# Enzymatic Glucose Fiber Sensor for Glucose Concentration Measurement with a Heterodyne Interferometry

**DOI:** 10.3390/s23062990

**Published:** 2023-03-09

**Authors:** Cheng-Chih Hsu, Wan-Yu Chung, Chun-Yi Chang, Chyan-Chyi Wu, Cheng-Ling Lee

**Affiliations:** 1 Department of Electro-Optical Engineering, National United University, No. 2 Lienda, Miaoli 36063, Taiwan; 2 Department of Photonics Engineering, Yuan Ze University, 135, Yuan-Tung Road, Taoyuan City 32003, Taiwan; 3 ASE Technology Holding Co., Ltd., Nantze Export Processing Zone, Kaohsiung 81146, Taiwan; 4 Department of Mechanical and Electromechanical Engineering, Tamkang University, New Taipei 25137, Taiwan

**Keywords:** enzymatic glucose sensor, no-core fiber, heterodyne interferometry

## Abstract

In this study, we developed a glucose fiber sensor incorporating heterodyne interferometry to measure the phase difference produced by the chemical reaction between glucose and glucose oxidase (GOx). Both theoretical and experimental results showed that the amount of phase variation is inversely proportional to glucose concentration. The proposed method provided a linear measurement range of the glucose concentration from 10 mg/dL to 550 mg/dL. The experimental results indicated that the sensitivity is proportional to the length of the enzymatic glucose sensor, and the optimum resolution can be obtained at a sensor length of 3 cm. The optimum resolution of the proposed method is better than 0.6 mg/dL. Moreover, the proposed sensor demonstrates good repeatability and reliability. The average relative standard deviation (RSD) is better than 10% and satisfied the minimum requirement for point-of-care devices.

## 1. Introduction

The Centers for Disease Control and Prevention (CDC) released the latest scientific data on diabetes in the United States in 2020, showing that about 10% (29.1 million) of the population of the United States has diabetes. The report also noted that the cost of diabetes-related treatment in the United States in 2012 was estimated at approximately $245 billion [1]. The International Diabetes Federation announced that diabetes continues to be a growing health burden in low- and middle-income countries, with 592 million people living with diabetes in 2035, twice as many as in 2013 [2]. Numerous clinical studies have confirmed that controlling lower blood glucose levels can reduce risk factors for cardiovascular disease, and that self-monitoring of blood glucose levels can be effectively performed using commercial blood glucose meters. Guidelines from the National Institutes of Health (NIH) recommend that patients with type 2 diabetes typically perform self-testing before meals, after meals, and before bedtime [1,3]. Therefore, measuring glucose concentration with a blood glucose meter is essential to reduce the costs associated with diabetes. 

Blood glucose concentration measurement methods can be divided into non-invasive methods [4,5,6] and invasive methods [7,8,9,10,11,12,13,14,15,16,17,18,19,20,21]. While invasive methods do not provide painless measurements, these methods can avoid individual patient differences (including race, skin color, skin composition, skin thickness, and complex blood components) and increase the reliability of clinical diagnosis. Therefore, currently available methods for clinical monitoring of blood glucose levels are based on the invasive (in vitro) methods. The invasive method can be categorized into electrochemical methods [7,8] and optical methods [9,10,11,12,13,14,15,16,17,18,19,20,21]. Many review papers [7,8] pointed out the advantages and challenges of the electrochemical method for blood glucose concentration monitoring. The most important issue with electrochemical methods is to reduce or eliminate calibration procedures while taking into account measurement accuracy to improve the convenience of measurement [7]. In contrast to electrochemical methods, optical methods [9,10,11,12,13,14,15,16,17,18,19,20,21] are measured non-contact. There is no contact between the electrode and the test piece as in electrochemical measurement, eliminating the calibration problems caused by contact measurement. In addition, due to the diversity of optical measurement methods, the design of the sensor can measure the concentration of the sample according to the change in the physical characteristics of the sample (such as refractive index (RI), polarization state, optical rotation power, light intensity, wavelength shift, etc.).

Lin [9] proposed a heterodyne refractometer to determine the refractive index and chiral parameter of the various concentration of glucose solution. Based on its optical configuration, when it is incident on the sample at critical angle, the phase difference of the interference signal will be discontinuous, and then the RI and the corresponding glucose concentration will be obtained. The results showed that the optimal resolution of RI could achieve 10^−5^. Bhardwaj et al. [10] developed double tapered Mach–Zehnder interferometer for RI measurement of glucose solution at different concentrations. Their results showed that the minimum concentration variation of glucose solution was 2%. Chiu et al. [11] developed a novel measurement method for small rotation power of glucose solution. To control the azimuth angle of a half-wave plate, the rotation power of glucose solution can be obtained. Their results showed that a wide measurement range of glucose concentration and the resolution of the rotation power can be better than 1.6 × 10^−5^ °/mm. Upadhyay et al. [12] proposed a double D-shaped fiber Bragg grating (FBG) for RI measurement of glucose concentration varied from 0% to 50%. Their results indicated that the sensitivity of double D-shaped FBG was better than that of D-shaped FBG, and the optimal sensitivity could reach 47.37 nm/RIU. Zhong et al. [13] proposed a glucose sensor based on helical intermediate period fiber grating (HIPFG) structure. The sensitivity of HIPEG glucose sensor was about 0.026 nm/(mg/mL) and the detection limit can achieve 1 mg/mL. Wu [14] fabricated s-shaped long period fiber grating (LPFG) and immobilized glucose oxidase (GOx) on the s-shaped LPFG surface for glucose concentration measurement. The measurement range of glucose concentration was covered 0 wt%–1 wt% with high linearity, and the sensitivity of Wu’s method could reach 6.229 dB/wt%. Azkune et al. [15] modified polymer fiber surface with phenylboronic acid (PBA) and Alizarin Red S(ARS) for glucose sensors. Based on the evanescent wave characteristics at the U-shaped fiber/sample interface, the transmitted light will be related to the sample concentration. Their method showed a detection limit of 0.1 M for glucose concentration. Hsu et al. [16] developed a circular heterodyne polarimeter and fabricated a reusable enzymatic glucose sensor for measuring glucose concentration. For glucose solutions, the repeatability and resolution of the proposed system were better than 95% and 0.88 mg/dL, respectively. The reusable glucose sensor could be reused consecutively 100 times for application, and it provided a similar response efficiency. Badmos et al. [17] developed an enzymatic long-period fiber grating (LPFG) glucose sensor. Based on LPFG’s dual sensing peaks, their method provided two measurement ranges for glucose concentration. The optimal linear measurement range was 0.1 mg/mL to 3.2 mg/mL with a detection wavelength of 1787 nm. Zhou et al. [18] modified 4-vinylphenylboronic acid (4-VPBA) on the surface of the helical long-period grating (HLPG) for glucose sensors. The linear range of the glucose concentration covered 0.18–3 mg/mL and preserved similar sensitivity over 3 weeks of the sensor. Lee et al. [19] fabricated a glucose sensor by constructing a long-period grating on a panda-type polarization maintaining (PM) fiber and immobilized GOx on the surface. The results showed that the transmitted intensity of linear horizontal polarization (LHP) was inversely proportional to the glucose concentration around the wavelength of 1548 nm. Compared to the results of LHP, the results of the linear vertical polarization (LVP) exhibited redshift as glucose concentration increased around the wavelength of 1606 nm. Zhang et al. [20] developed an MSM-SPR sensor with the structure of multimode fiber/single-mode fiber with surface plasmon resonance structure/multimode fiber for glucose concentration measurement. MSM-SPR sensor combined with a glucose emzymatic reaction device consisting of GOx modified polystyrene (GOx-PS) and MnO_2_ can detect the RI variation of gluconic acid at different glucose concentrations. Results showed that the glucose enzymatic reaction device could be reused 10 times without significant difference. Previous work [21] fabricated an enzymatic fiber sensor by immobilizing GOx on the core surface of a single mode fiber (SMF) to measure the glucose concentration in human serum. Results showed that the sensor could be reused 13 times within 1 week, and the theoretical resolution could be better than 0.15 mg/dL.

To increase the number of applications of fiber type glucose sensor, this study optimized the technology of GOx immobilized on the no-core fiber surface. Therefore, the new glucose sensor can be reused nearly three times more than the sensor produced by the previous work [21]. The glucose sensor had a single mode fiber (SMF)/enzymatic no-core fiber/single mode fiber structure, and the results showed that the measurement sensitivity was proportional to the length of the enzymatic no-core fiber. Two ends of the SMF adopted FC type bare fiber adapter to easily replace the glucose sensor. Based on the optical configuration of the proposed system, the phase variation could achieve a phase stability of 0.07° in 30 s. Therefore, theoretical resolutions were about 1.154 and 0.577 mg/dL for sensors with lengths of 1 cm and 3 cm, respectively. These findings suggest that the proposed sensors and measuring devices can serve as alternative systems for in vitro clinical examinations and become green clinical diagnostic systems for long-term care centers in future.

## 2. Principles

**Figure 1:**
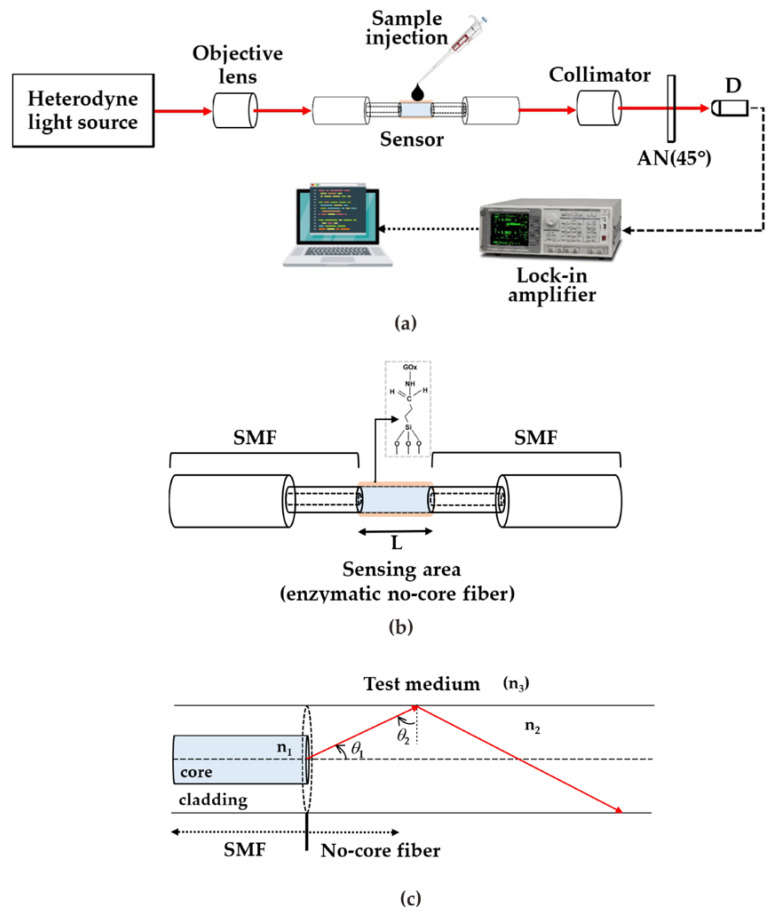
(**a**) Diagram of the experimental setup; (**b**) sensor structure; and (**c**) TIR phenomenon at boundary between no-core fiber and test medium.

Figure 1a shows the measurement setup of the proposed method. The heterodyne light source was generated by an electro-optical modulator with 1k Hz modulation frequency. The heterodyne light was guided into the enzymatic glucose sensor constructed with enzymatic no-core fiber with single-mode fiber (SMF) spliced at both ends. The two ends of the SMF are with FC type bare fiber adaptor. The diagram of the sensor structure is shown in Figure 1b. 

When light is incident into the enzymatic no-core fiber at an angle of *θ*_1_ through a single-mode fiber, total internal reflection (TIR) will occur at the boundary between the enzymatic no-core fiber and the test medium. Therefore, the phase shift between p- and s- polarizations can be obtained and written as [22]

(1) $$\delta =\delta_{p}-\delta_{s}=2{tan}^{-1}\left(\frac{\sqrt{{sin}^{2}\theta_{2}-n^{2}}}{tan\theta_{2}sin\theta_{2}}\right)$$

where n=n3n2 indicated the ratio between the refractive indices of no-core fiber (*n*_2_) and test medium (*n*_3_); $\theta_{2}=90{}^{\circ}-\theta_{1}$, which is shown in Figure 1c. Depending on the numerical aperture (NA) of objective lens and the refractive index (*n*_1_) of SMF, the maximum value of *θ*_1_ will be in the range of 4° to 9°. Obviously, the light beam will exhibit multiple TIR in no-core fiber and the number of TIRs can be expressed as

(2) $$m=\frac{L}{2d\cdot tan\theta_{2}}$$

where L and *d* are the length and diameter of no-core fiber, respectively. Based on the Jones calculation [23], the interference signal detected by detector (**D**) can be written as

(3) $$I_{t}=\frac{1}{2}\left[1+cos\left(\omega t+m\delta \right)\right]=\frac{1}{2}\left[1+cos\left(\omega t+\varphi \right)\right]$$

The total phase shift (*ϕ*) can be obtained immediately by a lock-in amplifier with phase-lock technology. When a sample is injected into the sensing area, glucose oxidase (GOx) catalyzes the conversion of glucose to gluconic acid and hydrogen peroxide [24]. Thus, the total phase shift (*ϕ*) of TIR changes as the chemical reaction progresses, and the heterodyne light carries this time-varying signal. Therefore, by measuring *ϕ* at different glucose concentrations, the calibration curve of phase versus glucose concentration can be obtained.

**Figure 2:**
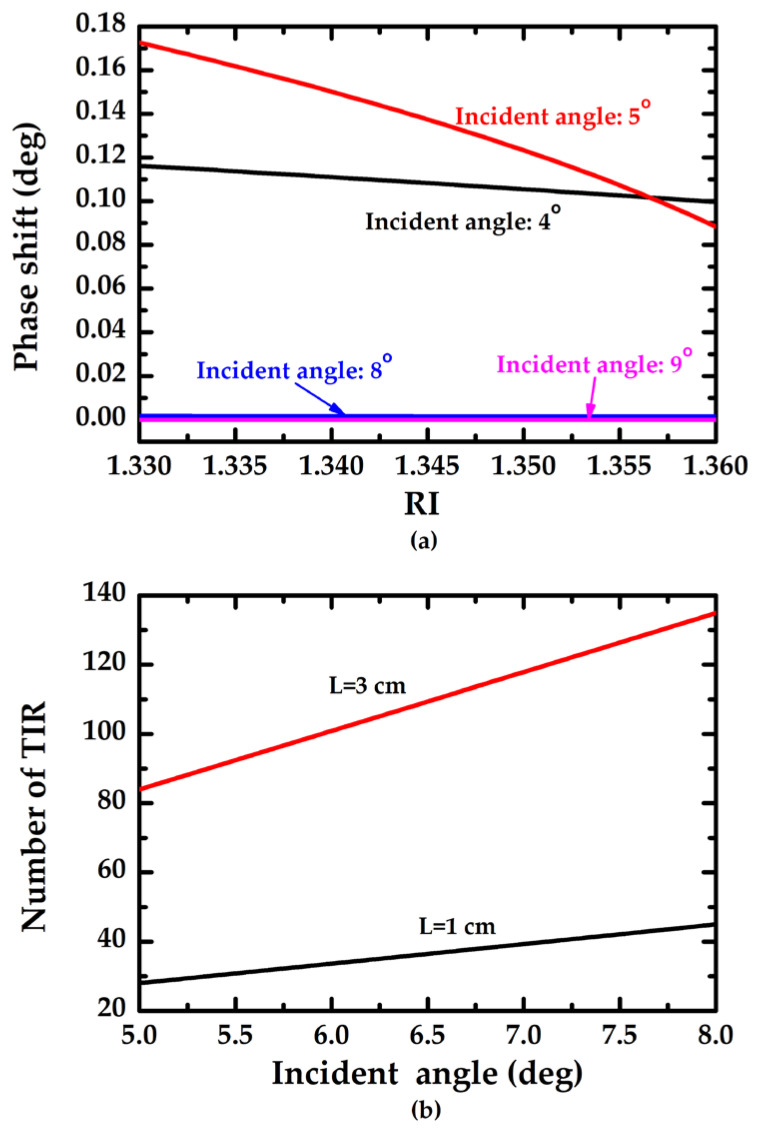
Theoretical simulation. (**a**) Phase shift vs. RI under various incident angle (*θ*_1_); (**b**) number of TIR vs incident angle (*θ*_1_) under various length (L) of enzymatic no-core fiber.

According to Equations (1) and (2), the relationship between the phase shift and refractive index (RI) of test medium can be seen in Figure 2a; the number of TIR and incident angle (*θ*_1_) can be seen in Figure 2b. Figure 2a shows that the phase shift is inversely proportional to RI of the test medium, and the optimum variation of phase shift will be approximated of 0.08° as *θ*_1_ = 5°. Figure 2b shows that the TIR number is proportional to the length of no-core fiber, and the longer the length of enzymatic no-core fiber (sensing area), the more times of TIR.

## 3. Experimental Results

### 3.1. Sensor Fabrication

**Figure 3:**
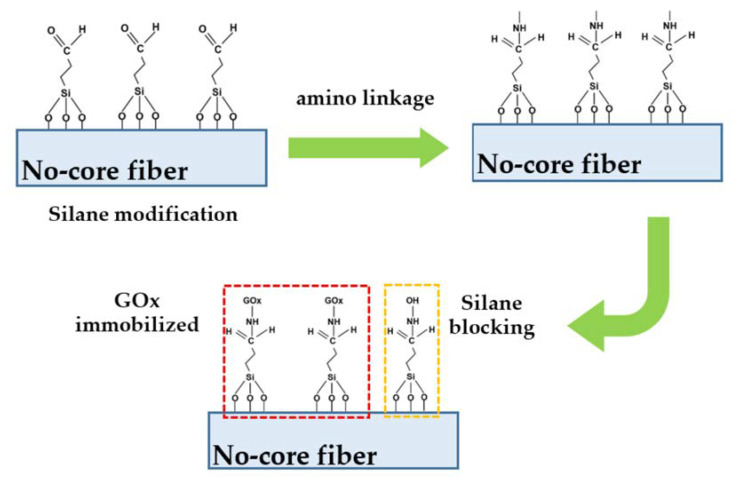
The fabrication procedures of enzymatic no-core fiber sensor.

The enzymatic glucose sensor was constructed with enzymatic no-core fiber with single-mode fiber (SMF). These fibers were purchased from commercially available manufactures where no-core fibers (Prime Optical Fiber Corporation, Hsinchu, Taiwan, model:NCF125) and SMFs (Thorlabs Inc., Newton, NJ, USA, model: SM600) have a cladding diameter of 125 μm. The numerical aperture of SMF is 0.1–0.14. The no-core fiber sensor was treated with 1% (*v*/*v*) 3-(trimethoxysilyl)propyl aldehyde in absolute ethanol for 30 min at room temperature. After washing with ethanol for few times, the fiber sensor was dried by N_2_ air gun, followed by heating at 100 °C for 45 min. To immobilize GO_x_ on SiO_2_ with covalent bonding via the amino linkage aldehyde group of fiber sensor surface, the modified surface was then covered with a 175 µg/mL GOx in 10 mM phosphate buffered saline (PBS) solution at pH 7 for 1 h. Unreacted aldehyde groups were quenched by immersion in 15 mM Tris buffer solution (pH 7.5) for 10 min at room temperature. The fabrication procedure is shown in Figure 3. 

**Figure 4:**
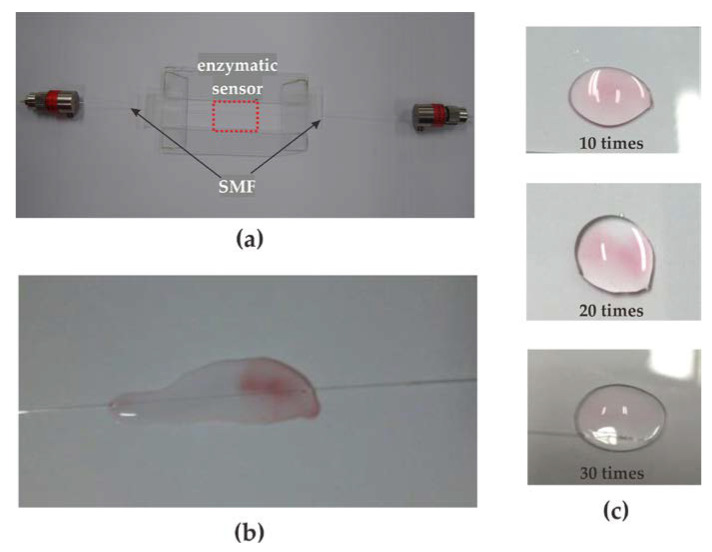
The pictures of the enzymatic no-core fiber sensor. (**a**) Photo of the sensor; (**b**) chromogen test result of GOx activity; (**c**) consecutive chromogen test for the reusability of sensor.

After fabrication of the enzymatic no-core fiber sensor, the effectiveness of GOx on the sensor surface will be verified using a standard validation method provided by the World Health Organization, (WHO) [25]. Glucose present in the test solution will be oxidized by GOx to form gluconic acid and hydrogen peroxide. Hydrogen peroxide will be converted to water and oxygen by peroxidase (POD). The oxygen acceptor 4-4-aminophenazinone absorbs oxygen and forms a pink chromogen together with phenol. Therefore, when the sensor was placed in a solution containing glucose, POD, and 4-4-aminophenazone, the activity of GOx on the sensor surface could be determined by the appearance or absence of pink color. In addition, for the convenience of sensor replacement, the two ends of the SMF are connected to the light source and detector with FC type bare fiber adaptor. As shown in Figure 4, Figure 4a shows the photo of the actual enzymatic no-core fiber sensor, and Figure 4b is the verification result of GOx activity. Figure 4c shows the reusable behavior by consecutive chromogen test and the darker the pink color, the higher the enzyme activity. Thus, the proposed sensor can be reused at least 30 times.

### 3.2. Performance of the Proposed Method

**Figure 5:**
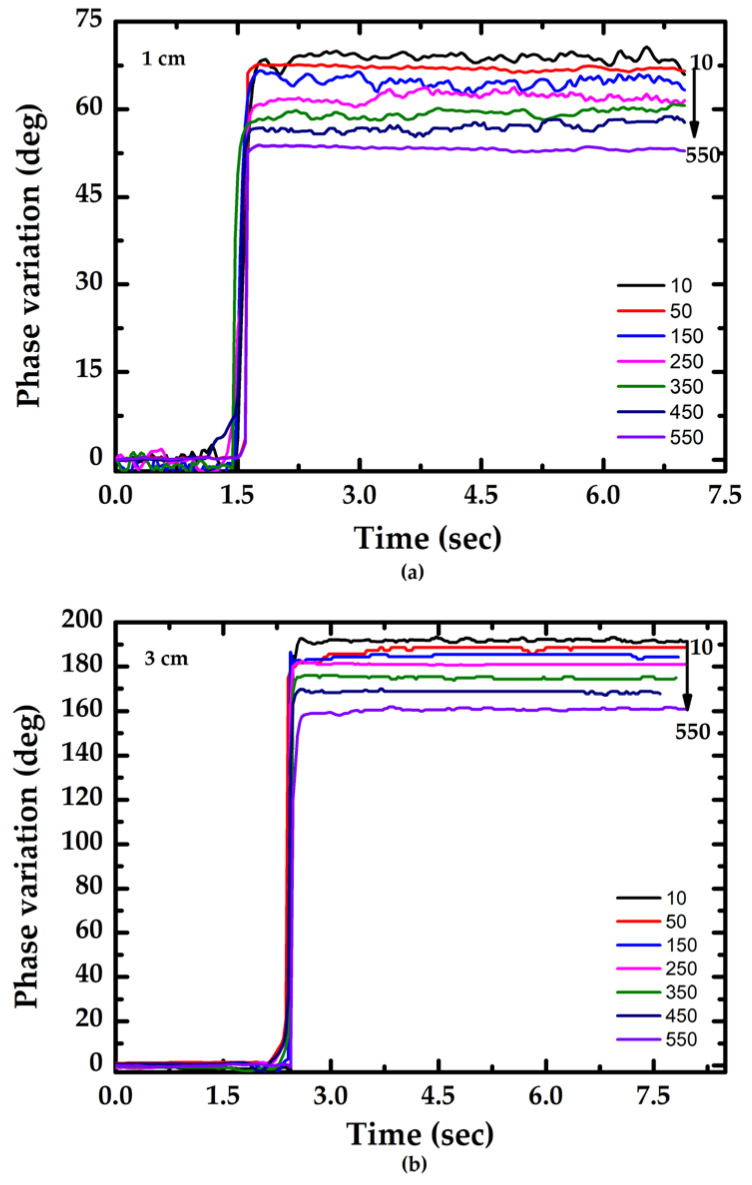
Phase–time response curves of the proposed sensor with various lengths: (**a**) L = 1 cm; (**b**) L = 3 cm.

Various concentrations of glucose were prepared to demonstrate the performance of the proposed method. In this study, glucose solutions were prepared by dissolving glucose anhydrous in DI (deionized) water, and the glucose concentration was within the range of 10–550 mg/dL. Figure 5 shows the phase–time response curves of the proposed sensor with various lengths of enzymatic no-core fiber. As the sample was injected onto the sensing area, GOx catalyzed glucose and changed the RI of test medium. Therefore, the total phase shift varied as the reaction progressed. The results showed that the phase variation was inversely proportional to the glucose concentration, and the reaction was terminated within 3 s. In general, the refractive index increases with the increase in glucose concentration. The simulation results in Figure 2a show that as the refractive index increases, the phase shift decreases. The experimental results are consistent with the theoretical predictions.

**Figure 6:**
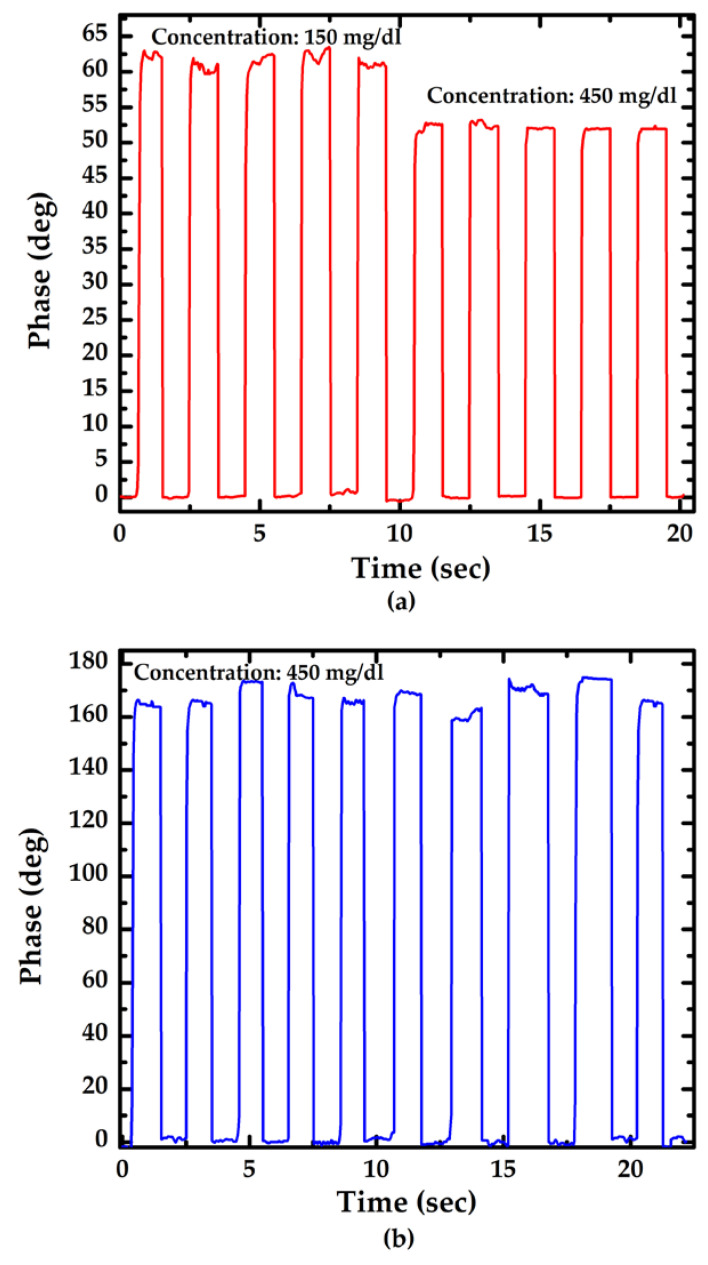
Repeatability evaluation of the sensor with various length of the sensor: (**a**) 1 cm; (**b**) 3 cm.

Figure 6 shows the results of consecutive tests of sensors with different lengths at various glucose concentrations. The glucose concentration of the test sample is controlled at 150 mg/dL and 450 mg/dL for the sensor with a length of 1 cm, and the glucose concentration of the test sample for the sensor with a length of 3 cm is controlled at 450 mg/dL. A test sample was injected with a volume of 1 cc directly with a micropipette and reacted with the sensor for 1–2 s. Next, the reacted liquid was aspirated out. In the consecutive testing, DI water was not used to clean the sensor. Terminal phase deviations can be caused by interference caused by improper aspiration of reactants, residual reactants on the sensor surface, and short sampling time frame. To prevent interference, the injection system can be replaced with an autosampler, which continuously injects sample and deionized water to clean the sensor surface.

In contrast to the consecutive testing, in a reliability test, the test sample reacted with the sensor for 10 s, the reacted liquid was sucked out, and then the sensor was cleaned twice with 10 cc of DI water. After each sensor has been used 10 times, it was replaced with another new sensor for the next set of 10 experiments. The reliability evaluation was performed by calculating the relative standard deviation (RSD) of 50 replicate experiments for each glucose concentration in accordance with Clarke’s method [26].

**Figure 7:**
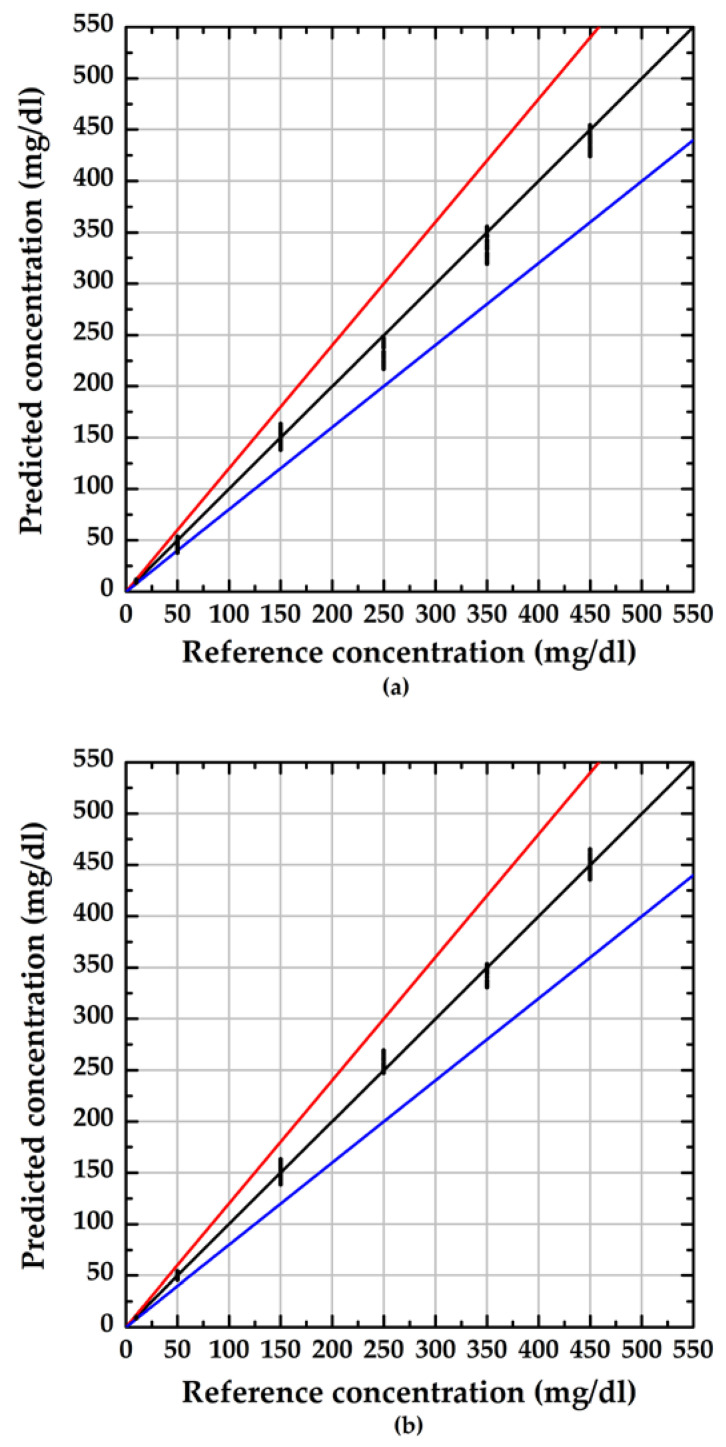
Reliability evaluation of the sensor with various length of the sensor: (**a**) 1 cm; (**b**) 3 cm.

The evaluation results are shown in Figure 7, and the values in the region between the red and blue lines indicate the measurement result within ±20% of the reference concentration. The average RSD of the proposed sensors with lengths of 1 cm and 3 cm were 9% and 6%, respectively. These results demonstrated that the proposed sensors have good reliability and met the minimum requirement (±15%) for point-of-care devices provided by US Food and Drug Administration (FDA) [27].

**Figure 8:**
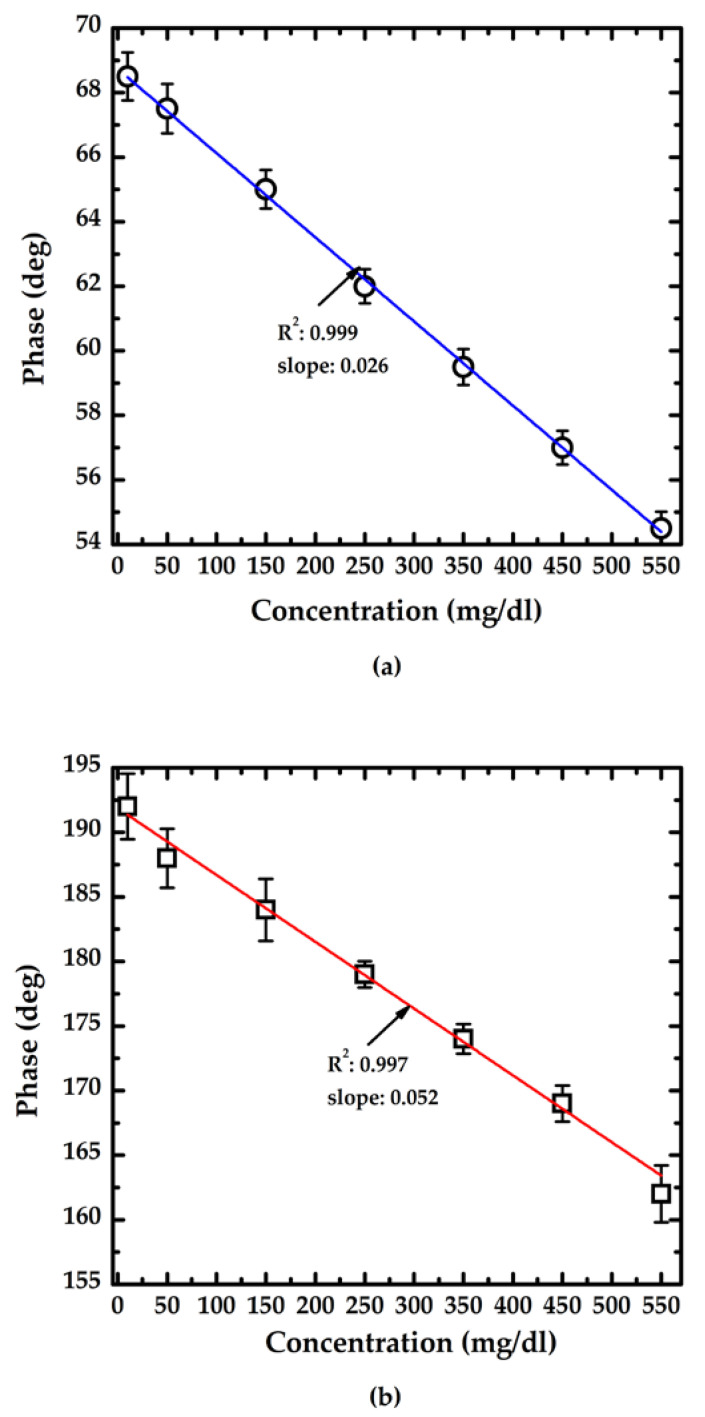
The calibration curves of the proposed method: (**a**) the sensor with length of 1 cm; (**b**) the sensor with length of 3 cm.

Figure 8 indicates that the calibration curve measured of the proposed method with various lengths of sensor. The symbols ◯, ☐, and I represent the average value of 10 measured data sets and the standard deviation of each concentration measured by the proposed sensor with 1 cm and 3 cm long sensors, respectively. The slope of the calibration curve indicates the sensitivity of the proposed method; the phase variation was approximately −0.026° for 1 mg/dL for the measurement by the sensor length of 1 cm and −0.052° for 1 mg/dL for the measurement by the sensor length of 3 cm. The shaper slope indicates a higher sensitivity, and obviously the sensor length of 3 cm provided higher sensitivity for glucose concentration measurement. The results show that the linear measurement range of the proposed method covers glucose concentrations from 10 to 550 mg/dL.

## 4. Discussion

Resolution Δ*C* of the proposed method can be achieved by calculating the ratio of the phase error |Δ*ϕ*| of the proposed method to the slope *S* of the calibration curve, which is expressed as

(4) $$\left|\Delta C\right|=\left|\frac{\Delta \varphi }{S}\right|$$

**Figure 9:**
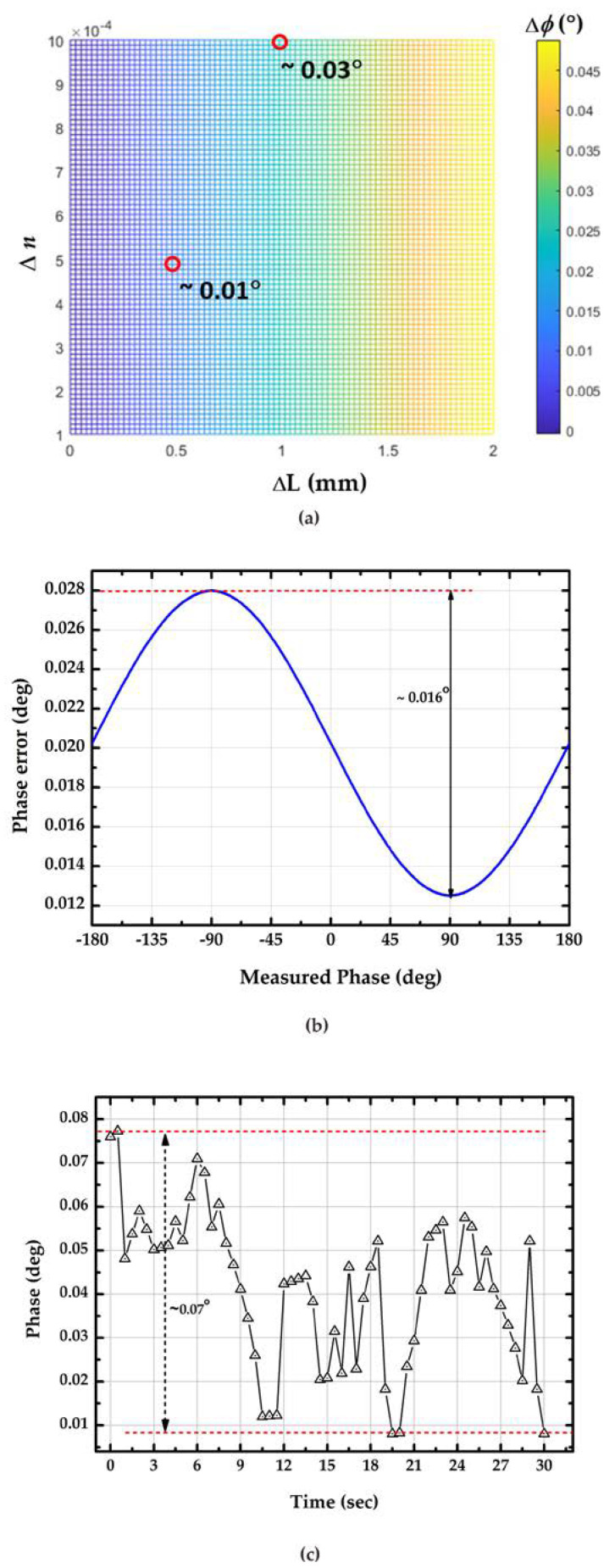
Phase error analysis of the proposed sensor. (**a**) Contribution of sensor length tolerance and variation of *n*; (**b**) residual phase error; (**c**) phase stability of the proposed method.

According to Equations (1) and (2), the phase error ∆*ϕ* is a function of length of enzymatic no-core fiber and the ratio between the refractive indices of no-core fiber and test medium, which can be derived and expressed as

(5) $$\begin{array}{l} \Delta \varphi =\frac{\partial \varphi (L,n)}{\partial L}\left|\Delta L\right|+\frac{\partial \varphi (L,n)}{\partial n}\left|\Delta n\right|\\ \begin{array}{cc} & =\frac{\delta }{2dtan\theta_{2}}\left|\Delta L\right|-\frac{nLsin\theta_{2}}{d\left({\left(tan\theta_{2}sin\theta_{2}\right)}^{2}+{sin}^{2}\theta_{2}-n^{2}\right)\sqrt{{sin}^{2}\theta_{2}-n^{2}}}\left|\Delta n\right| \end{array} \end{array}$$

where |ΔL| and |Δ*n*| are the errors of the length of enzymatic no-core fiber and the ratio between the refractive indices of no-core fiber and test medium, respectively. The reason for |ΔL| may be that the length fails to be precisely controlled when cutting the no-core fiber and |ΔL| can be less than 2 mm. The source of |Δ*n*| may be an improper temperature control around the test sample and sensor, as well as laser wavelength stability. Theoretically, within 1 °C of temperature change, |Δ*n*| is less than 0.001. The simulation result was shown in Figure 9a. When |ΔL| is 1 mm and |Δ*n*| is 0.001, |Δ*ϕ*| is 0.03°. If |ΔL| is controlled at 0.5 mm and the temperature is controlled within 0.5 °C, |Δ*n*| will be within 5 × 10^−4^, and therefore |Δ*ϕ*| will be close to 0.01°. With reference to the analysis of Wu [28], the residual nonlinearity phase error was evaluated, and the results indicate that phase error was less than 0.02°, which is shown in Figure 9b. Based on the error analysis and considering |ΔL|, |Δ*n*|, and residual nonlinearity phase error, the theoretical phase error |Δ*ϕ*| can be better than 0.03°.

Unfortunately, imperfect temperature control of the solution, unexpected electronic variations, and the residual nonlinearity periodic error of the measurement apparatus also affected the phase error of the proposed method. The practical phase error of the proposed method is indicated by evaluating the phase stability of the measurement system, and the results are shown in Figure 9c. The practical phase error of the proposed system was 0.07° within 30 s. Based on the error analysis, the resolution of the proposed method can be estimated using Equation (4), and it is summarized in Table 1. If only residual phase error is considered as the phase error of the proposed system, the optimum resolution of the glucose solution can reach 0.615 and 0.308 mg/dL for the sensor lengths of 1 cm and 3 cm, respectively. According to the concept of detection limit (DL) proposed by Barrios et al. [29,30], |Δ*C*| can be regarded as the DL of the proposed method. Therefore, for glucose solution samples, the DL of the proposed method can be better than 3 mg/dL. Interferences in blood samples (e.g., L-ascorbic acid, methylmalonic acid, glycine, urea, etc.) may affect the accuracy of glucose concentration measurement. Wu et al. [31] evaluated the effect of these interferents on an optical glucose sensor with a GOx sensing layer. Their results showed a difference of less than 2.3% between the measurement of glucose concentration in samples with and without interference. Therefore, those potential interferents may not have a significant impact on the accuracy of the proposed sensor.

Table 2 summarizes the performance comparison results between the proposed sensor and the related work cited in Section 1. The proposed method yields a reusable glucose sensor with acceptable detection limits, fast response time, and wide measurement range. 

## 5. Conclusions

This work demonstrates the feasibility of an enzymatic glucose sensor and integrates into a heterodyne interferometry for measuring the glucose concentration. Experimental results indicate that the resolution of the proposed sensor is strongly related to the length of the enzymatic sensor. The resolution increases with increasing sensor length, and the optimum resolution is approximately 0.577 mg/dL. Moreover, the limit of detection of the proposed method is approximately 1.346 mg/dL. This work further demonstrates the repeatability of the proposed sensor, maintaining an acceptable phase–time response under 10 consecutive uses for sensors of different lengths. Importantly, the proposed method is highly promising for its repeatability and reliability required by the FDA. Based on these findings, a reusable and reliable enzymatic sensor was fabricated and integrated into an apparatus with high sensitivity and a high-resolution system for glucose concentration measurements. We hope that the proposed system can be used as a green clinical diagnostic system in long-term care centers.

## Figures and Tables

**Table 1:** The resolution of the proposed method with different sensor length.

L	|*S*| (°/(mg/dL))	Theoretical		Practical	
		Δ*ϕ* (°)	|Δ*C*| (mg/dL)	Δ*ϕ* (°)	|Δ*C*| (mg/dL)
1 cm	0.026	0.03	1.154	0.07	2.692
3 cm	0.052		0.577		1.346

**Table 2:** Performance comparison of relevant methods.

Ref.	Detection Limit	Linear Range	Response Time	Reusability	Method	Enzyme Adopted
[9]	Better than 50%	X	X	X	Polarimeter	X
[10]	2%	0%–10%	X	X	Double tapped Mach-Zehnder interferometer	X
[11]	Better than 10 mg/dL	10–1000 mg/dL	X	X	Polarimeter	X
[12]	10%	0%–50%	X	X	Double D-shaped FBG	X
[13]	1 mg/mL	0.02–200 mg/mL	X	X	HIPFG	X
[14]	0.25 wt%	0 wt%–1 wt%	X	X	S-shaped LPFG	GOx
[15]	0.1 M	X	10 min	X	U-shaped fiber	PBA-ARS
[16]	1.41 mg/dL	1–450 mg/dL	<10 s	>100 times	Circular polarimeter	GOx
[17]	X	0.1–3.2 mg/mL	X	X	LPFG	GOx
[18]	0.037 mg/mL	0.18–3 mg/mL	X	X	HLPG	4-VPBA
[19]	5 mM	5–25 mM	X	X	PM-LPFG	GOx
[20]	X	20–400 mg/dL	~8 min	10 times	SPR fiber	GOx
[21]	0.139 mg/dL	SRM 965a [32]	<2 s	13 times	SMF	GOx
This work	1.346 mg/dL	10–550 mg/dL	<3 s	30 times	No-core fiber with heterodyne interferometry	GOx

## Data Availability

Not applicable.
